# Low genetic diversity in broodstocks of endangered Chinese sucker, *Myxocyprinusasiaticus*: implications for artificial propagation and conservation

**DOI:** 10.3897/zookeys.792.23785

**Published:** 2018-10-23

**Authors:** Dongqi Liu, Yu Zhou, Kun Yang, Xiuyue Zhang, Yongbai Chen, Chong Li, Hua Li, Zhaobin Song

**Affiliations:** 1 Sichuan Key Laboratory of Conservation Biology on Endangered Wildlife, College of Life Sciences, Sichuan University, Chengdu 610065, PR China; 2 School of Biological and Chemical Engineering, Panzhihua University, Panzhihua 617000 , PR China; 3 China Three Gorges Corporation, Beijing 100038, PR China; 4 Fisheries Research Institute, Sichuan Academy of Agricultural Sciences, Chengdu 611731, PR China; 5 Key Laboratory of Bio-Resources and Eco-Environment of Ministry of Education, College of Life Sciences, Sichuan University, Chengdu 610065, PR China

**Keywords:** genetic management, genetic varieties, *
Myxocyprinus asiaticus
*, parent fish, resources protection

## Abstract

The releasing program of Chinese sucker (*Myxocyprinusasiaticus*) has been conducted for years in China. To prevent loss of genetic variation in wild populations, it is important to assess and monitor genetic diversity of broodstocks before release of offspring. Three broodstocks (Pixian Base of Sichuan Fisheries Research Institute, China (PBS), Yibin Base of Sichuan Fisheries Research Institute, China (YBS) and Yibin Rare Aquatic Animal Research Institute, China (YRA)) were investigated using mitochondrial control region and 12 microsatellites. The relatively low genetic diversities of these broodstocks were detected (PBS, haplotype diversity (h) = 0.877, observed heterozygosity (Ho) = 0.416; YBS, h = 0.812, Ho = 0.392; YRA, h = 0.818, Ho = 0.365). PBS showed higher Ho than YBS and YRA (*P <* 0.05). Genetic divergence (F_ST_) based on microsatellites between PBS and YRA was significant (F_ST_ = 0.1270, *P* < 0.05), the same situation happened between YBS and YRA (F_ST_ = 0.1319, *P* < 0.05). However, divergence between PBS and YBS was not significant (F_ST_ = 0.0029, *P* > 0.05). Structure analysis revealed that YRA were distinct from PBS and YBS. Based on these results, it is important to propose some suggestions of genetic management for artificial propagation of Chinese sucker, such as broodstock exchange among hatcheries and broodstock supplement from wild.

## Introduction

*Myxocyprinusasiaticus* (Nelson, 1976), an endangered freshwater fish in China and the only representative of the family Catostomidae in Asia (Nelson 1976; Wang 1998), is distributed mainly in the Yangtze River drainage (Ding 1994). It used to be an important part of fish catches in its distribution areas (Zhang et al. 2000; Zhu et al. 2009). But since 1970s, natural reproduction and resources of *M.asiaticus* have dramatically declined due to some anthropogenic factors, such as habitat destruction, water pollution, and over fishing (Zhang et al. 1999, 2000; Zhang and Zhao 2000; Jiang and Yu 2003; Gan et al. 2011). Therefore, *M.asiaticus* was listed as second class national protected animal in China (Wang 1998; Wang and Xie 2004).

In order to restore the wild resources in the Yangtze River drainage, artificial propagation of *M.asiaticus* has been carried out since the 1970s, and a releasing program on a large scale was conducted first in 1996 (Zhang et al. 2000; Zhu et al. 2009). Release of hatchery reared individuals may increase the productivity of fishery, accelerate recovery of depleted stocks, and ensure the survival of stocks threatened with extinction (Ireland et al. 2002). But without genetic management, genetic variation of the hatchery juveniles will be reduced, which may have negative impacts on wild populations after releasing (Gow et al. 2011; Ortega-Villaizan et al. 2011). An effective restocking program for endangered fish populations requires not only the increase of quantity, but also a broad recovery of their genetic diversities (Vrijenhoek et al. 1985). For example, studies on genetic management of *Huchohucho* and *Salvelinusalpinus* have been launched in Europe or North America (Blackie et al. 2011; Kucinski et al. 2015). However, compared to nearly 20-year history (since 1996) of artificial breeding and releasing of *M.asiaticus*, genetic management studies on broodstocks are very limited. The genetic investigation has only been carried out in a few broodstocks of *M.asiaticus* (Xu et al. 2013), and the genetic management has so far almost not been considered.

Because wild individuals were difficult to obtain in recent years and the qualified parental fish used in propagation were limited, some of first generation offspring of *M.asiaticus* were supplemented as broodstocks for artificial propagation (Wu 2014). Besides, there were almost no detailed archives of each individual in those broodstocks, so effective management could not be executed and genetic variation of the hatchery stock may be reduced. The situation would be more serious after several generations of artificial propagation.

**Figure 1.**
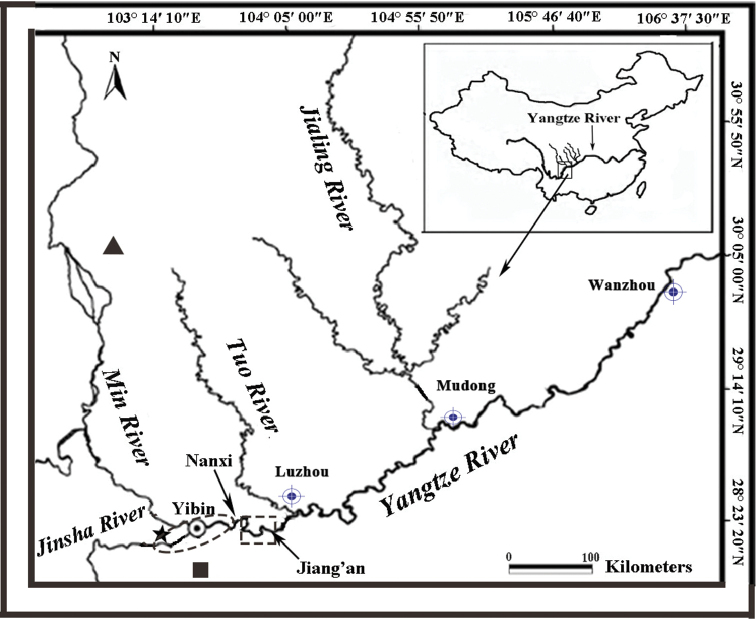
Map of sampling sites for the three *Myxocyprinusasiaticus* broodstocks. Key: black triangle Pixian Base of Sichuan Fisheries Research Institute, China (PBS); black star Yinbin Base of Sichuan Fisheries Research Institute, China (YBS); black square Yibin Rare Aquatic Animal Research Institute, China (YRA); broken circle river range where the wild broodstocks source of YBS; broken rectangle river range where the wild broodstocks source of YRA.

Wu (2014) reported the genetic diversity of three wild populations (Wanzhou, Mudong, and Luzhou; Figure 1) based on mtDNA control region and microsatellites. The genetic diversity of broodstocks needs to be compared with that of wild populations, which is very important for artificial propagation and wild conservation of the species. Previous studies based on microsatellite markers reported low genetic diversity and weak differentiation in broodstocks of *M.asiaticus*, which were mainly collected from middle Yangtze River (Xu et al. 2013). However, as the major participator of releasing program in upper Yangtze River, broodstocks in Sichuan Province have not been investigated and managed systematically yet. It was far from enough for genetic analysis of Chinese sucker broodstocks. In this study, by means of mitochondrial control region and 12 microsatellite markers, we present a genetic study of three broodstocks of *M.asiaticus* from different local hatcheries in Sichuan. Our objectives are (i) to assess the genetic diversity and relationship of the broodstocks; (ii) to propose suggestions of genetic management for artificial propagation, and (iii) to provide necessary information for genetic conservation for implementation hatchery release program in the future.

## Materials and methods

### Sample collection

A total of 134 individuals of *M.asiaticus* were used in this study, including 53 (15 males and 38 females) sampled from the Yibin Base of Sichuan Fisheries Research Institute, China (**YBS**), 60 (22 males and 38 females) from the Pixian Base of Sichuan Fisheries Research Institute, China (**PBS**) and 21 (7 males and 14 females) from the Yibin Rare Aquatic Animal Research Institute, China (**YRA**) (Fig. 1). Fin clips of each individual were sampled and stored in 95% ethanol during 2013 – 2015. Some individuals of YBS are wild ones captured from the Yibin range of the Jinsha River, and from Yibin and Nanxi range of Yangtze River in middle of 1990s (Figure 1) and most are first generation offspring of wild *M.asiaticus* that were artificially propagated and reared in YBS for more than ten years. Some individuals of PBS are first generation offspring of wild *M.asiaticus* artificially propagated in YBS and reared for more than ten years as well. The rest individuals of PBS are wild, but their sources are unclear. All individuals of YRA are wild ones captured from Nanxi and Jiang’an range of the Yangtze River (Figure 1) in middle of 1990s.

In addition, the artificially propagated Chinese suckers were firstly released into the Yangtze River in 1996. The released juveniles were much smaller than the wild originated individuals when they were captured from the rivers. Therefore, it is not likely that wild collected broodstocks included some artificially released ones or hybridized ones of artificially breeding broodstock and wild populations.

### DNA extraction and PCR amplification

Total genomic DNA was extracted from the fin clips of *M.asiaticus* using TIANamp marine animals DNA Kit (TIANGEN, China). Polymerase chain reaction (PCR) was used to amplify the mitochondrial control region with the primers DL1 (ACCCCTGGCTCCCAAAGC, Ta: 61^o^C) and DH2 (ATCTTAGCATCTTCAGTG, Ta: 61^o^C) (Liu et al. 2002). The PCR protocol followed Wu et al. (2016). PCR products were purified using E.Z.N.A. Gel Extraction Kit (OMEGA, USA) and then directly sequenced on an ABI 3730 Genetic Analyzer (Applied Biosystems, China).

Twelve microsatellite loci (Yang et al 2009; Chen et al 2010) were used for analyses. The total PCR reaction volume was 25 μL contained 1 μL genomic DNA (50 ng/μL), 3 μL 10×PCR buffer (plus Mg^2+^), 1 μL dNTPs (10 mmol/L each), 0.5 μL for each primer (10 μmol/L), 0.5 U Taq DNA polymerase (TaKaRa, Japan), and 18.7 μL ddH_2_O. PCR was programmed as follows: 94 °C for 5 min, followed by 35 cycles at 94 °C for 30 s, 54–63 °C for 40 s, 72 °C for 30 s, and a final extension at 72 °C for 10 min. Electrophoresis, fluorescent microsatellite detection and determination of genotypes were as described in Chen et al. (2010).

### Data analysis

All mitochondrial control region sequences were aligned using Mega version 5.0 (Kumar et al. 2004) and refined manually. Nucleotide composition, number of polymorphic sites (v), haplotype diversity (h) and nucleotide diversity (π) (Nei 1987) were used to evaluate the genetic diversity of samples, and were estimated by DNASP version 4.10.7 (Rozas et al. 2003). We used two methods to construct the phylogenetic trees of the haplotypes: maximum likelihood method (ML) by PAUP version 3.1.1 (Swofford 2002) and Bayesian inferences (BI) by MRBAYES version 3.1.2 (Huelsenbeck and Ronquist 2001). Control region sequences of *Catostomuscommersonii* (GenBank No. AB127394) and *Cycleptuselongates* (GenBank No. EF062437) were obtained from GenBank and used as outgroups. The relationships among the haplotypes were evaluated by NETWORK version 4.2.0.6 (Bandelt et al. 1999). Genetic distances among broodstocks were calculated by MEGA version 5.0 (Kumar et al. 2004).

To estimate genetic diversity of the three broodstocks and genetic differentiation among them, numbers of alleles (**A**), allelic richness within individuals (**Ai**), expected and observed heterozygosities (**Ho** and **He**) were calculated using the software AUTOTET which is especially developed for autotetraploid species (Thrall and Yong 2000; Seeber et al. 2014; Hopley et al. 2015; Liu et al. 2015; Qiang et al. 2015). The genetic diversities of the broodstocks were compared with that of the wild populations investigated by Wu (2014) and the broodstocks investigated by Xu et al. (2013). Paired *t*-test was used to evaluate whether significant differences of diversity indices occurred among populations (Jansson et al. 2012). The number of rare alleles and private alleles were calculated by Convert version 1.31 (Glaubitz 2004). Exact tests for Hardy–Weinberg equilibrium and tests for linkage disequilibrium were conducted using GENEPOP version 4.0 (Raymond and Rousset 1995). Null alleles were tested in MICROCHRCHER version 2.2 (Oosterhout et al. 2004). To evaluate the amount of genetic variation among and within broodstocks, an analysis of molecular variance (AMOVA) was conducted in ARLEQUIN version 3.11 (Excoffier et al. 2005). Pairwise F_ST_ were also calculated in ARLEQUIN version 3.11. F_IS_ were calculated for polymorphic loci using FSTAT version 2.932 (Goudet 2002). A test for bottleneck assessment was conducted using the BOTTLENECK version 1.9 (Piry et al. 1999). Neighbor-joining tree of broodstocks and relationship of individuals was constructed in MEGA version 5.0. Clustering procedure was performed to infer the relationship of broodstocks in STRUCTURE version 2.3 (Pritchard et al. 2000). We set the number of clusters (K) to vary initially from 1 to 8 (ten replicates for each K). Each run started with a burn-in period of 100,000 steps followed by 1,000,000 Markov Chain Monte Carlo (MCMC) steps. Finally, the Delta K method in STRUCTURE HARVESTER (Earl et al. 2012) was used to infer the optimal K value.

## Results

### Genetic diversity

Control region sequences for 134 individuals of *M.asiaticus* were acquired and the aligned sequences were 947 base pairs (bp) in length. The number of variable sites was 82 (71, 68, 65 in PBS, YBS, YRA, respectively). Most polymorphic sites were transitional mutations, and only a few were transversions or inserts/deletions. The average nucleotide difference was 24.1%. The average base composition was A = 28.5 %, T = 31.4 %, C = 22.4 % and G = 17.7 %. The haplotype diversity and nucleotide diversity was 0.864 and 0.028 across all samples, respectively (Table 1). PBS showed higher haplotype diversity (h = 0.877) than that of YBS and YRA (h = 0.812 and 0.818, respectively) (Table 1). However, YRA possessed higher nucleotide diversity (0.0278) than that of PBS and YBS (Table 1).

Among the 12 microsatellites, one locus (MA61) was monomorphic in all broodstocks, two (MA53, MA39) were monomorphic in PBS and YBS, and one (MA21) was monomorphic in YRA. The number of amplified alleles per locus ranged from 1 (MA61) to 16 (MA27) with an average of 8.1, and allele richness per locus varied from 1.00 at locus MA61 in PBS to 2.00 at locus MA64 in YBS (Table 2). Average observed heterozygosity (Ho) ranged from 0.365 in YRA to 0.416 in PBS. Under both chromosome segregation and chromatid segregation, average values of Ho were lower than those of expected heterozygosities (He) in all broodstocks (Table 2), and average values of the fixation index estimated over all loci were positive in all broodstocks analyzed, suggesting a deficit of heterozygotes in all broodstocks. Total 29 rare alleles and 19 private alleles were identified in the three broodstocks. The number of rare alleles ranged from 8 to11, and the number of private alleles ranged from 2 to 13 (Table 1). YRA possesses most private alleles (13) and almost half rare alleles (11) (Table 1).

The broodstocks from middle Yangtze River were investigated based on same microsatellites, and Average He ranged from 0.443 to 0.523 (Xu et al. 2013), which were higher than that of the three broodstocks in the present study (paired *t*-test, *P* < 0.05). Wu (2014) reported the genetic diversity of three wild populations (Wanzhou, Mudong, and Luzhou) based on mtDNA control region and same microsatellites. Wanzhou showed higher haplotype diversity (h = 0.975) than that of Mudong and Luzhou (h = 0.905 and 0.899, respectively). Average PIC ranged from 0.779 in Luzhou to 0.816 in Wanzhou. Compared to wild populations of *M.asiaticus* (Wu 2014; Wu et al. 2016), lower genetic diversities (Ai, G, Ho, He, PIC) in the broodstocks were found in present study (paired *t*-test, *P* < 0.05).

**Table 1.** Information of the three *Myxocyprinusasiaticus* broodstocks and genetic diversity.

Broodstock	N	n	h	π	Ra	Pa	F_IS_	*P*
PBS	60	19	0.877	0.0260	8	4	0.055	0.421
YBS	53	13	0.819	0.0238	10	2	0.067	0.390
YRA	21	11	0.818	0.0278	11	13	0.088	0.085
Total	134	30	0.864	0.0287	29	19		

N: sample number, n: number of haplotypes, h: haplotype diversity, π: nucleotide diversity, Ra: rare alleles for microsatellites, Pa: private alleles for microsatellites, FIS: mean inbreeding coefficients for microsatellites, P: the p value of FIS

**Table 2.** Genetic diversity of the *Myxocyprinusasiaticus* broodstocks based on twelve microsatellite loci.

Broodstock	Locus	A	Ai	G	Ho	He(Ce)	He(Cd)	PIC
PBS	MA21	5	1.933	13	0.622	0.732	0.683	0.684
	MA10	6	1.650	11	0.433	0.623	0.582	0.575
	MA61	1	1.000	1	0.000	0.000	0.000	0.000
	MA06	2	1.567	3	0.378	0.420	0.392	0.331
	MA19	7	1.867	17	0.578	0.818	0.764	0.793
	MA53	1	1.000	1	0.000	0.000	0.000	0.000
	MA39	1	1.000	1	0.000	0.000	0.000	0.000
	MA64	3	1.983	3	0.656	0.516	0.481	0.398
	MA04	9	1.717	20	0.478	0.816	0.762	0.791
	MA38	3	1.600	5	0.400	0.584	0.545	0.519
	MA27	16	2.683	35	0.758	0.902	0.842	0.894
	MA13	10	2.333	19	0.683	0.824	0.769	0.805
	Means	5	1.694	11	0.416	0.520	0.485	0.483
YBS	MA21	4	1.900	8	0.600	0.726	0.678	0.676
	MA10	5	1.833	7	0.556	0.664	0.620	0.602
	MA61	1	1.000	1	0.000	0.000	0.000	0.000
	MA06	2	1.367	3	0.244	0.406	0.379	0.323
	MA19	7	1.733	9	0.489	0.699	0.652	0.646
	MA53	1	1.000	1	0.000	0.000	0.000	0.000
	MA39	1	1.000	1	0.000	0.000	0.000	0.000
	MA64	2	2.000	1	0.667	0.500	0.467	0.398
	MA04	7	1.833	9	0.556	0.681	0.636	0.375
	MA38	3	1.633	5	0.422	0.531	0.496	0.519
	MA27	13	2.733	15	0.761	0.888	0.829	0.877
	MA13	5	1.633	9	0.406	0.676	0.631	0.609
	Means	4	1.639	6	0.392	0.481	0.449	0.419
YRA	MA21	1	1.000	1	0.000	0.000	0.000	0.000
	MA10	6	1.710	10	0.476	0.718	0.670	0.386
	MA61	1	1.000	1	0.000	0.000	0.000	0.000
	MA06	6	1.760	10	0.508	0.782	0.730	0.431
	MA19	3	1.140	4	0.095	0.534	0.498	0.384
	MA53	2	1.710	2	0.476	0.459	0.429	0.321
	MA39	8	1.810	10	0.540	0.756	0.706	0.598
	MA64	3	1.950	4	0.635	0.618	0.577	0.378
	MA04	3	1.000	3	0.000	0.594	0.554	0.711
	MA38	6	1.760	10	0.508	0.724	0.676	0.739
	MA27	7	2.000	6	0.667	0.811	0.757	0.788
	MA13	3	1.710	4	0.476	0.582	0.543	0.711
Mean		4	1.550	5	0.365	0.548	0.512	0.454

A: number of alleles per locus, Ai: allelic richness within individuals, G: genotype richness, Ho: observed heterozygosity, He (Ce): expected heterozygosity under chromosome segregation, He (Cd): expected heterozygosity under chromatid segregation, PIC: polymorphism information content

### Genetic relationship

**Figure 2.**
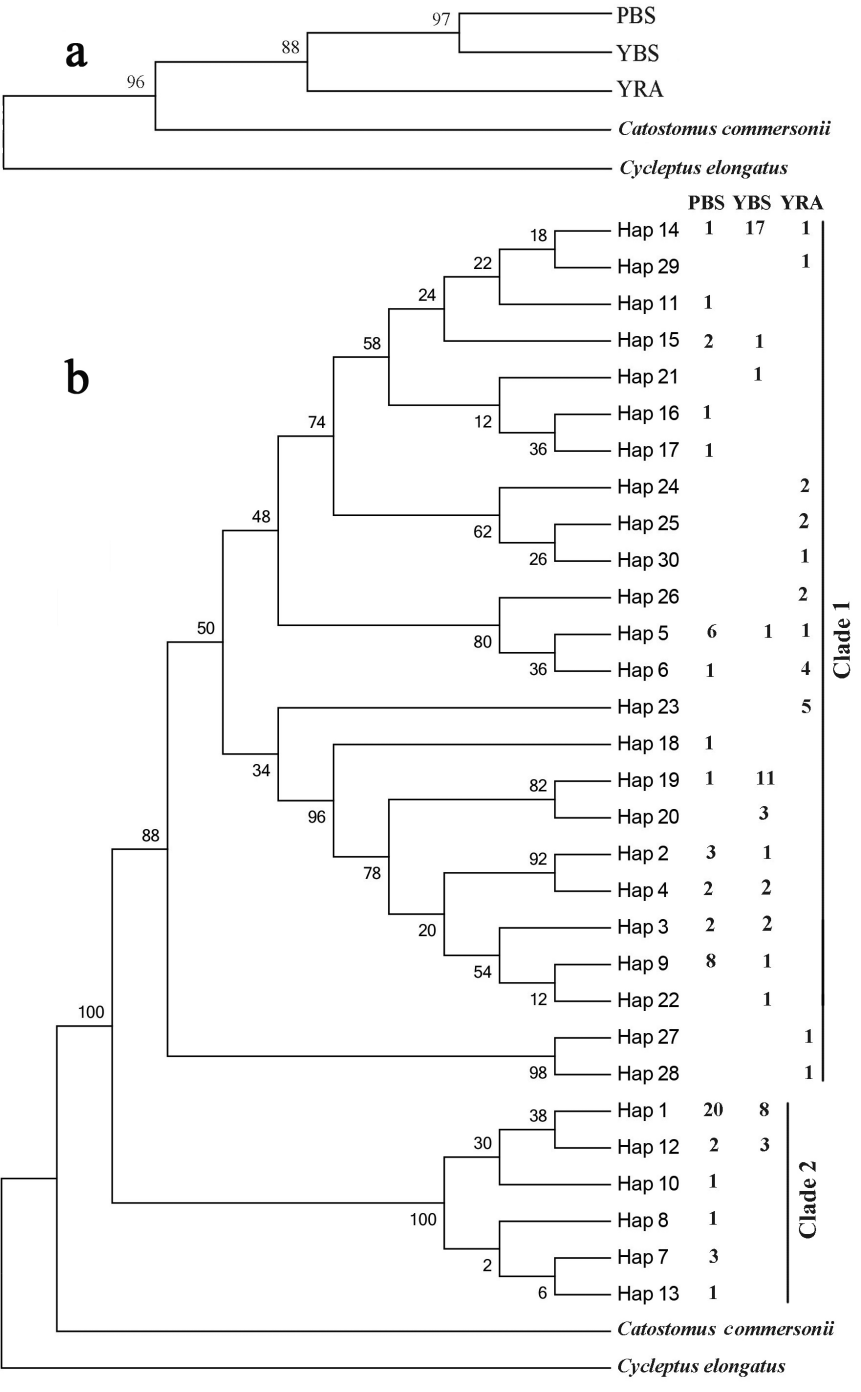
Neighbor-joining tree based on F_ST_ values of the mtDNA control region (**a**) and phylogenetic tree of mtDNA control region haplotypes in *Myxocyprinusasiaticus* reconstructed with Bayesian inference (**b**). Bayesian posterior probabilities and bootstrap values are shown at nodes of neighbor-joining tree and BI tree, respectively. The number behind each haplotype represents the number of individuals from different sampling locations.

An AMOVA performed based on microsatellite markers showed insignificant molecular variance among broodstocks (6.45%, *P* > 0.05) and significant variance among individuals within hatcheries (93.55 %, *P* < 0.01). Significant divergence was observed between PBS and YRA (F_ST_ = 0.1270, *P* < 0.05), and the same situation occurred between YBS and YRA (F_ST_ = 0.1319, *P* < 0.05). But no significant divergence was observed between PBS and YBS (F_ST_ = 0.0029, *P* > 0.05). The analysis based on mtDNA control region showed congruent results derived from microsatellites. The genetic distances between PBS and YRA, YBS and YRA were larger than that between PBS and YBS. Furthermore, PBS and YBS broodstocks were clustered together in neighbor-joining tree based on F_ST_ values (Figure 2a).

**Figure 3.**
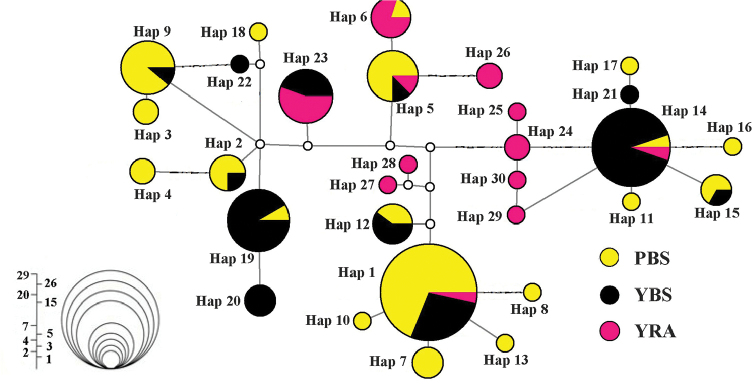
Median-joining network of the mtDNA control region haplotypes of *Myxocyprinusasiaticus*. The size of each circle indicates the frequency of the corresponding haplotype in the whole data set.

The topologies of the phylogenetic trees produced by ML and BI were nearly identical (Bayesian tree was presented in Figure 2b). All mtDNA haplotypes were clustered into two distinct clades that were well supported by high bootstrap values. Clade 1 was composed of the haplotypes from all three broodstocks, while clade 2 was composed of the haplotypes only from PBS and YBS. Only two of the 30 haplotypes (Hap14, Hap5) were shared among all three samples while 8 haplotypes were shared between PBS and YBS, and most haplotypes in YRA were clustered together. Hap1 was most widespread, and included 28 individuals (Figure 2b). The median-joining network of all haplotypes showed that distribution of haplotypes from PBS and YBS were widespread, but the YRA haplotypes were more concentrated. Hap1, hap14, hap19, and hap9 were the most common haplotypes. PBS and YRA possessed ten and seven specific haplotypes, respectively and YBS had only three (Figure 3).

**Figure 4.**
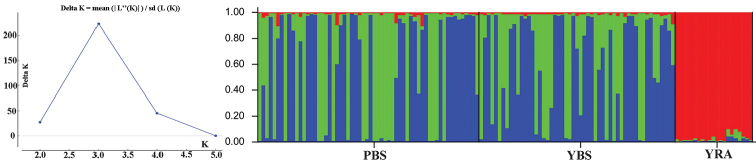
Results of STRUCTURE of *Myxocyprinusasiaticus* broodstocks based on K = 3. Each column represents one individual and the colors represent the probability membership coefficient of that individual for each genetic cluster.

Structure Harvester online showed the highest peak of Delta K (222.58) when K = 3 (Figure 4), which indicated that the genetic structure of the broodstocks had three genetic clusters. The majority of YRA broodstock belonged to one cluster, and most of individuals from PBS and YBS broodstocks were promiscuously assigned to the other two clusters, which suggested that YRA were distinct from PBS and YBS (Figure 4).

**Figure 5.**
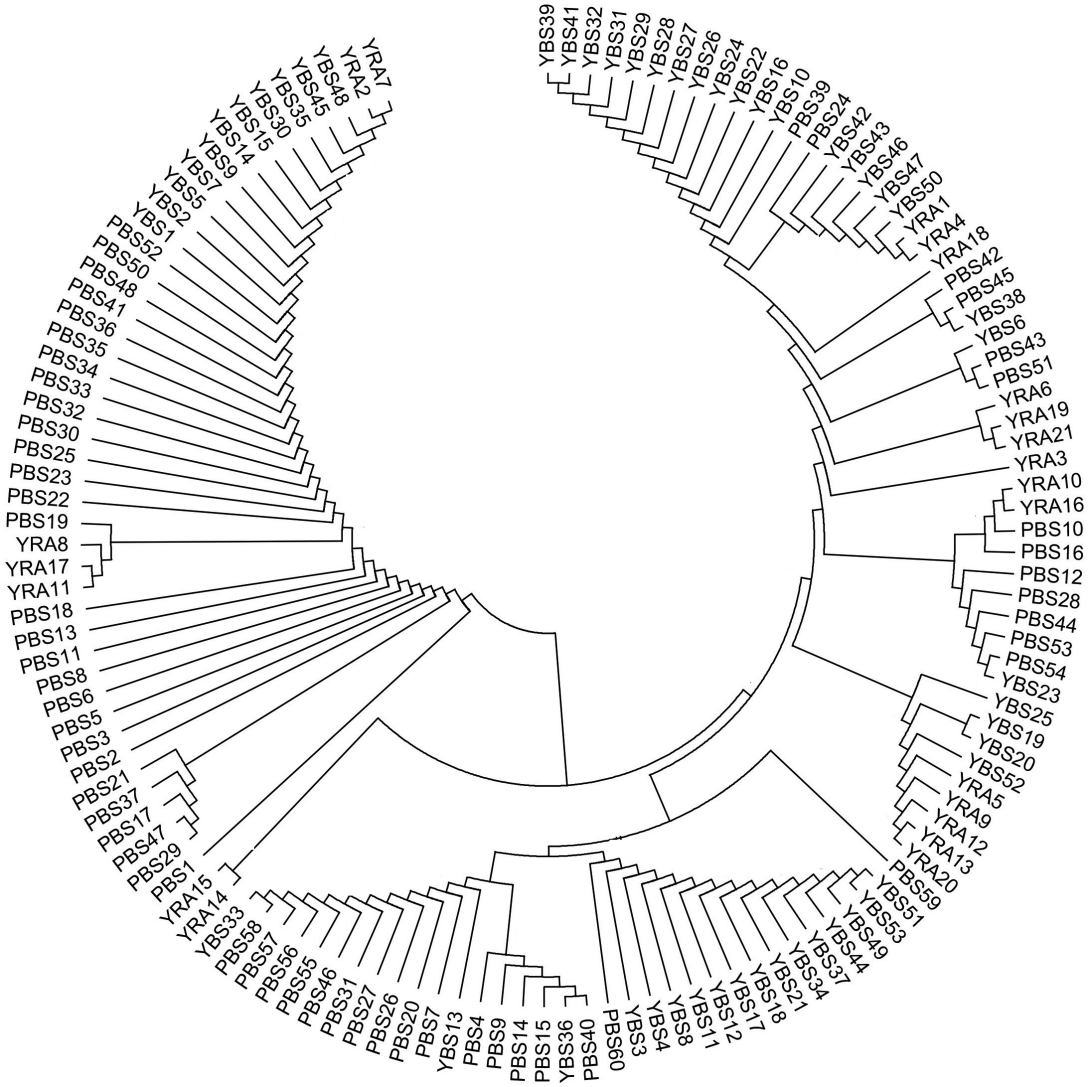
Neighbor-joining trees of individuals in three *Myxocyprinusasiaticus* broodstocks based on mtDNA control region.

On the basis of individual genetic distance, individual neighbor-joining tree was divided into two main branches, with one of them further divided into two branches. Individual distributions were widespread in neighbor-joining tree and did not cluster together based on broodstocks (Figure 5).

### Demography of broodstocks

The F_IS_ was calculated using 12 microsatellite loci. Insignificant F_IS_ was found in all broodstocks (*P* > 0.05). Under the infinite allele model (IAM) heterozygosity excesses were detected in YBS and YRA. However, under stepwise mutation model (SMM) and the two-phase model (TPM), all broodstocks showed insignificant heterozygosity excess, and did not suffer from bottleneck or founder effects in the past (normal situation in Wilcoxon sign rank test) (Table 3).

**Table 3.** Probabilities from tests (Wilconxon’s) for mutation drift equilibrium (bottlenecks) in the three *Myxocyprinusasiaticus* broodstocks under three mutation models (IAM, TPM and SMM).

Broodstocks	Mutation-drift test			Model shift
	I.A.M	S.M.M	T.P.M	L-shaped
PBS	0.1243	0.5431	0.3330	normal
YBS	0.0465*	0.6772	0.7870	normal
YRA	0.0374*	0.3448	0.0625	normal

* p < 0.05 (rejection of mutation drift equilibrium)

## Discussion

Through surveys and interviews of the three hatcheries before, we knew that the hatcheries reared a limited number of parental fish. Furthermore, those parental fish which could be qualified for artificial propagation were fewer. Besides, some individuals of first generation of artificially propagated *M.asiaticus* have been used as parental fishes and usually, one male was used for propagation with five or more females. These contributed to the lower genetic diversities in the broodstocks than that in wild populations of *M.asiaticus* (Wu 2014; Wu et al. 2016) and broodstocks investigated by Xu (2013) which used the different set of microsatellites and consequently might resulted in inbreeding and thus reduced the genetic quality of the offspring easily.

Both microsatellites and mtDNA markers revealed that the genetic diversity of YRA was lower than that of YBS and PBS, which might be attributed to the relative small sample size of YRA, and there were not enough wild ones to supplement broodstocks for a long time. In addition, both Pixian Base and Yibin Base hatcheries belong to the Sichuan Fisheries Research Institute, and thus frequently exchange the parental fish each other. This should contribute the relatively higher genetic variations in these two stocks as well.

Private alleles were results of lengthy evolution, which may have some special adaptation function or chain with some special properties (Zhang 2008). Both private and rare alleles have important values in propagation and may be lost from gene pool in domestic environment due to the role of genetic drift (Zhang 2008). Therefore, it is important to preserve these rare and private alleles of *M.asiaticus* broodstocks during artificially propagating, especially for YRA. YRA possesses most private alleles and almost half rare alleles. Nevertheless, the numbers of rare and private alleles might be affected by the number of parental fish in each hatchery, and should be further investigated through relatively large samples of broodstocks in the hatcheries in order to provide more accurate data for propagation in the future.

A previous study revealed that there was insignificant genetic differentiation in most broodstocks of *M.asiaticus* (Xu et al. 2013). The present study also showed insignificant genetic differentiation between YBS and PBS. The genetic distance was very low (0.002) between them. However, the genetic differentiations between PBS and YRA, and between YBS and YRA were significant. The genetic distances between YRA and PBS or YBS were larger than that between PBS and YBS. Great genetic differences between YRA and PBS or YBS might be attributed to that they originated from different cohorts in upper Yangtze River. Also, it was probably caused by a small number of wild founders which possessed limited gene pool and easily resulted in genetic drift (Xu et al. 2013). According to our investigation, some individuals we sampled from the PBS came from the first generation of offspring of YBS, which therefore weakened the genetic differentiation between them. There are more haplotypes shared between PBS and YBS than between one of them and YRA. Structure analysis indicated that some individuals from PBS and YBS belonged to one cluster. Therefore, PBS and YBS of *M.asiaticus* have a very close genetic relationship, and YRA was a separated broodstock.

According to the analysis of genetic diversity and relationship of the three broodstocks, we can propose some implications for artificial propagation and releasing program of *M.asiaticus*. First, although hatchery-release program has not much affected the genetic diversity (Wu 2014; Wu et al. 2016) and inbreeding coefficient and bottleneck of the three broodstocks were not significant, potential inbreeding problems are likely in the future. So the hatcheries should pay attention to the individual genetic relationship during artificial propagating and improve aquaculture conditions to increase the number of qualified individuals. The fish should be fed with more animal foods rather than ordinary pellet fodder, flowing water should be provided to stimulate broodstocks before propagation rather than still water environment, sufficient light and dissolved oxygen, and optimum temperature should be kept during over-wintering stage to avoid death and accelerate development, and disease prevention and control, especially for saprolegniasis and parasite infection, should be paid more attention to as well. In order to increase size of qualified broodstocks and replenish the genetic pool, hatcheries should make efforts to collect more wild mature individuals. Second, breeding without genetic management will exacerbate decreasing genetic diversity and may increase genetic divergence among cultured broodstocks of different hatcheries, which may finally affect the wild populations (Gow et al. 2011; Ortega-Villaizan et al. 2011). Unfortunately, according to the results of the present study, genetic diversity and relationship of the broodstocks seemed to have been artificially influenced. Restocking efforts should strive to maintain genetic connectivity and exchange among local broodstocks in the hatcheries to avoid inbreeding and increase genetic variation. Especially, many rare and private alleles in YRA might have important values in propagation or adaption, so it is necessary to do genetic exchange between YRA and PBS or YBS. Because the broodstocks of three hatcheries originated from neighboring ranges of the Yangtze River, genetic exchange does not mean mixing different genetic populations and would not affect the genetic characteristics of the wild populations through hatchery-release program. Hatcheries also need to record all details of each individual including body weight and length, gender, ID and genetic information. This precise information of existing broodstocks will be helpful for effective management (Kucinski et al. 2015). Third, in order to make sure that individuals are better adapted to live in natural environment, the juveniles of each batch should be randomly sampled for examining. Only when the samples are strong and healthy, can they be qualified for releasing. In addition, the continuous genetic monitoring of hatchery stocks is essential and baseline genetic data including archives and exchange records need to be renewed timely in the future, which are crucial to guide future population specific conservation programs and research efforts on *M.asiaticus* in China.
